# CLAffinity: A Software Tool for Identification of Optimum Ligand Affinity for Competition-Based Primary Screens

**DOI:** 10.1021/acs.jcim.2c00285

**Published:** 2022-04-20

**Authors:** Steven Shave, Nhan T. Pham, Manfred Auer

**Affiliations:** School of Biological Sciences, University of Edinburgh, The King’s Buildings, Edinburgh, Scotland EH9 3BF, United Kingdom

## Abstract

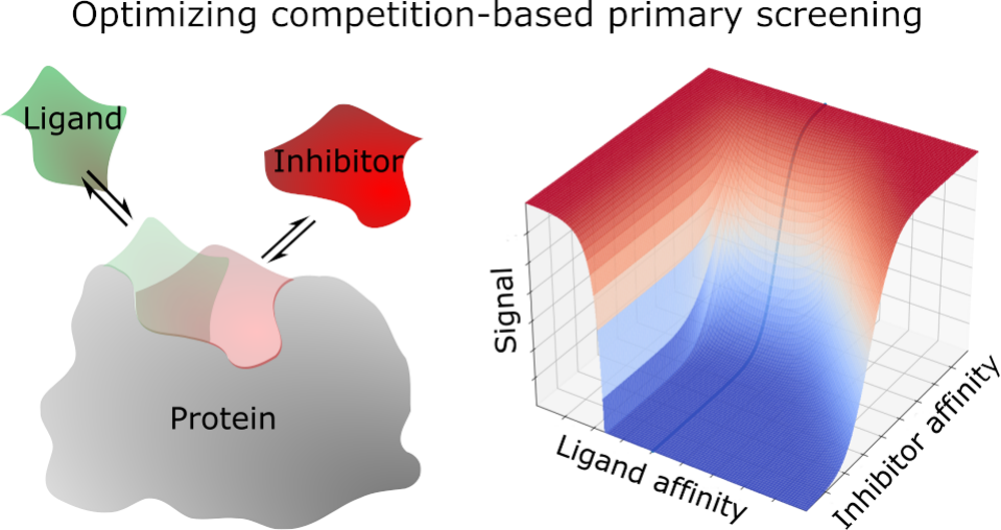

A simplistic assumption in setting up a competition assay is that a low affinity labeled ligand can be more easily displaced from a target protein than a high affinity ligand, which in turn produces a more sensitive assay. An often-cited paper correctly rallies against this assumption and recommends the use of the highest affinity ligand available for experiments aiming to determine competitive inhibitor affinities. However, we have noted this advice being applied incorrectly to competition-based primary screens where the goal is optimum assay sensitivity, enabling a clear yes/no binding determination for even low affinity interactions. The published advice only applies to secondary, confirmatory assays intended for accurate affinity determination of primary screening hits. We demonstrate that using very high affinity ligands in competition-based primary screening can lead to reduced assay sensitivity and, ultimately, the discarding of potentially valuable active compounds. We build on techniques developed in our PyBindingCurve software for a mechanistic understanding of complex biological interaction systems, developing the “CLAffinity tool” for simulating competition experiments using protein, ligand, and inhibitor concentrations common to drug screening campaigns. CLAffinity reveals optimum labeled ligand affinity ranges based on assay parameters, rather than general rules to optimize assay sensitivity. We provide the open source CLAffinity software toolset to carry out assay simulations and a video summarizing key findings to aid in understanding, along with a simple lookup table allowing identification of optimal dynamic ranges for competition-based primary screens. The application of our freely available software and lookup tables will lead to the consistent creation of more performant competition-based primary screens identifying valuable hit compounds, particularly for difficult targets.

## Introduction

Sensitive, parallelizable, and miniaturized competition experiments are commonly used in the primary screening phase of early drug discovery which aims at identification of hit compounds from, usually, medium to large sized libraries of small molecules.^1−5^ Exemplarily, a standard setup may begin with a target protein incubated with a fluorescently labeled ligand of known affinity. A signal such as fluorescence anisotropy^6^ may then be measured and correlated to the abundance of a protein–ligand complex. Small molecules contained in screening libraries are termed inhibitors for their potential to inhibit the protein–ligand interaction by competing to occupy the same shared binding site on a target protein. To be clear with our used naming conventions, we refer to “ligand” as the probe molecule from which a readout is taken, relating to its bound or unbound status (e.g., a fluorescently labeled small molecule, peptide, or similar), and “inhibitor” to be a molecule with the potential to compete with and inhibit this protein–ligand interaction by occupying a shared binding site. Screening library compounds are added at a common concentration of 10 μM. Higher concentrations around 50 μM may be more appropriate for fragment screens if solubility permits. These compounds are added either to the labeled ligand solution before a target protein is added or to the preformed complex after a certain incubation time to ensure equilibrium is achieved. With an increase or decrease of the signal from an instrument transformed into a measurement of the fraction ligand in complex with the target protein, dissociation of the protein–ligand complex caused by the inhibitor competing for the same binding site as the free labeled ligand can be monitored. Setting up competition experiments in this way uses the labeled ligand as a probe, through which the behavior of potential inhibitors in a screening library can be inferred, assuming both molecules bind exclusively to the same site on the target protein. A naive assumption in planning these screening assays would be that maximum sensitivity is achieved with a weakly binding ligand which is easy to compete with. However, characteristics of the competition system lead to practical considerations including instrument detection limits and the need for efficient use of reagents, making this assumption incorrect. The target protein and labeled ligand complex formation at a 1:1 stoichiometry is dependent on the concentration of protein present, the fundamental dissociation constant of the interaction, and the concentration of the labeled ligand. Increasing protein concentration and keeping a fixed concentration of the labeled ligand produce a hyperbolic binding curve with the complex concentration asymptotic to a maximum value. If we were determined to increase the fraction ligand bound (complexed ligand versus free ligand) at a given affinity, in order to produce a larger readout change on the addition of an inhibitor, we may consider increasing the ligand concentration while keeping the protein concentration constant. In addition to risking detector saturation or entering a nonlinear response range for the detector, this approach increases the amount of the free unbound ligand more than the bound ligand. Alternatively, increasing the protein concentration while holding the labeled ligand constant brings with it its own disadvantage; the abundance of free protein not bound to the ligand from which the signal is derived as a function of complex formation also increases. Inhibitor binding to free protein results in no detectable readout change, leading to a reduction in assay sensitivity. These primary screening assays assume complete equilibrium of the binding system. While the impact of incomplete equilibrium on competition experiment readouts is well documented,^7−9^ the complex characteristics and interaction between ligand affinity and competition assay sensitivity are best described at equilibrium.

A highly cited drug discovery technology paper by Huang provides a breakdown of the above considerations^10^ and even goes further to state that “a fluorescent ligand of highest affinity” should be selected for competition experiments. Clearly, using high affinity ligands leads to a reduction in the amount of protein needed to achieve the required fraction ligand bound in the absence of an inhibitor (see Supporting Information Figure S1). However, it also becomes harder for a competitive inhibitor to displace it. We have encountered firsthand misinterpretations of Huang’s advice, which is given in the context of competition experiments being used to determine inhibitor *K*_D_ values, rather than in the context of primary screens. Primary and secondary screening assays have different objectives, with the first optimized to give a clear yes/no indication of binding and prioritizing these compounds for secondary follow-up assays attempting accurate *K*_D_ value determination. No primary screening assay can stand alone and typically sit in a pipeline of orthogonal assays designed to exploit assay characteristics to minimize cost and effort. Full understanding of the characteristics and behavior of this first primary screening step impacts the entire pipeline. In a perfect assay setup in which even the lowest affinities may be detected, there are no false negatives. A hit compound can only appear as a false negative in the context of experimental variability and noise affected by assay sensitivity.

To examine the impact of following Huang’s advice without further investigation of the parameters of the assay planned for primary screening, we turned to simulation.

## Experimental Methods

Simulations were programmed and performed in Python^11^ (v3.7.1), making use of our already derived functions present in the PyBindingCurve^12^ package for simulation, fitting, and analysis of protein–ligand binding systems at equilibrium. These functions, specifically, direct analytical solutions to a 1:1:1 competition, were included in our open source tool CLAffinity, which is publicly available from a GitHub repository (https://github.com/stevenshave/competition-label-affinity) housing open source software for simulation and interrogation of competition-based primary screening systems or installable from the Python package index via the pip tool. The code and examples found in the GitHub source repository can be used to replicate all calculations, plots, and animations exhibited in this manuscript, as well as the accompanying Supporting Information and video.

To examine if Huang’s assertion to always use the highest available affinity ligand for *K*_D_ value determination also applies to primary screening, we set constraints and used conditions often applied in high-throughput screens.^13^ These are as follows: (i) to achieve a detectable signal, we assume the ligand is present at a concentration of 10 nM and labeled with a dye excitable at a common wavelength/laser line (e.g., 543 or 633 nm) with a reasonably strong quantum yield. This 10 nM labeled ligand concentration is typically found in fluorescence-based techniques and defined by equipment and physical limitations. (ii) We further assume that a strong “fraction ligand bound” signal is achieved when 70% of the ligand binds the protein in the absence of an inhibitor. All observations and conclusions drawn are valid at different percentages of a target ligand bound which may be more appropriate depending on the type of instruments, detection technologies, brightness, and photostability of dyes used in the experiments of any screening lab. For example, a 70% fraction ligand bound may be appropriate for fluorescence anisotropy-based techniques, but fluctuation analysis techniques^14^ working at a single molecule resolution, like fluorescence cross-correlation spectroscopy^15,16^ (FCCS) and two-dimensional fluorescence intensity distribution analysis,^17,18^ run in anisotropy mode (2D-FIDA-r), and TR-FRET readout systems may allow working with sub-10% fraction ligand bound assay systems.^19^

For any given ligand *K*_D_ value, we may calculate the required protein concentration to achieve a given fraction ligand bound (see Supporting Information eq S1). The direct analytical solution for a 1:1:1 competition (protein:ligand:inhibitor) is taken from our PyBindingCurve software, where rearrangement of mass balances resulted in a third order polynomial describing the system. One polynomial root is never physically relevant and can be discarded. From the remaining two roots, one is correct when the *K*_D_ value of the ligand is less than that of the inhibitor, and the other is correct when it is greater.

The Python functions defining the target fraction ligand bound and 1:1:1 competition make use of the MPMath^20^ (v1.1.0) library for arbitrary precision arithmetic, ensuring numerical stability and accuracy are retained even when dealing with large magnitudes of differences in concentrations and *K*_D_ values. Additionally, Numpy^21^ (v1.19.3) is used extensively to perform array operations, and Matplotlib^22^ (v3.3.2) is used to generate plot graphics and animations.

## Results and Discussion

**Figure 1:**
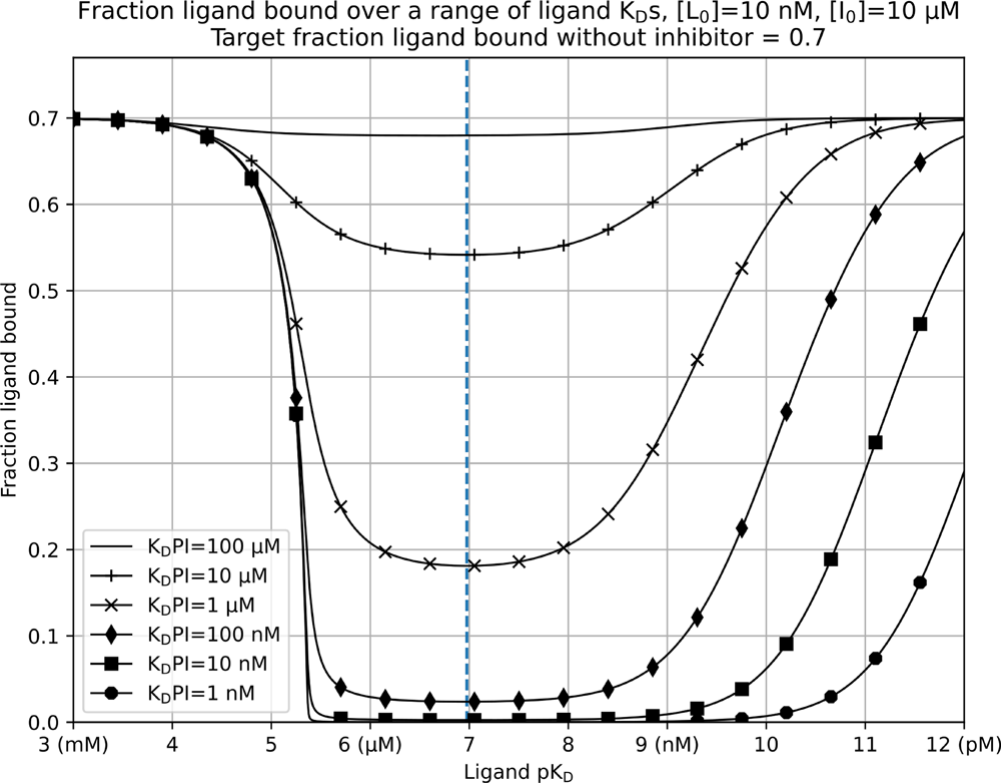
Demonstration of ligand affinity in competition experiments affecting the detection of inhibitors over a range of *K*_D_ values. Protein concentration calculated to obtain a 0.7 fraction ligand bound in the absence of an inhibitor. *K*_D_PI is protein-inhibitor complex affinity, [*L*_0_] is the total ligand concentration, and [*I*_0_] is the total inhibitor concentration. The maximum signal by deviation from a 0.7 fraction ligand bound for all inhibitor affinities in this example system is denoted by the dashed vertical line and achieved with ligand p*K*_D_ of 6.975 (105 nM *K*_D_).

Pharma companies routinely screen compound archives against targets at a standard concentration of 10 μM^18^ and a target fraction ligand bound of ∼0.7. Knowing these values, we simulated the fraction ligand bound (readout) when inhibitors of varying target protein affinities are introduced. These simulations are visualized in Figure 1, with the *x*-axis representing the labeled ligand *K*_D_ value expressed as p*K*_D_ (−log_10_(*K*_D_)) and transitioning from low to high affinity (*x*-axis). This produces a characteristic “valley” response for the fraction ligand bound. At lower affinities with p*K*_D_s up to 7 (*K*_D_ > 100 nM), we see evidence for Huang’s argument to always use the highest affinity ligand available, even here in primary screening. With increasing labeled ligand affinity, less target protein is required to achieve a 0.7 fraction ligand bound. The assay becomes more sensitive, and a larger drop in the signal for a given inhibitor affinity is detected. However, as ligand affinity is further increased beyond p*K*_D_s of 7 (*K*_D_ < 100 nM), the dynamic range of the assay response is reduced by difficulty in displacing the labeled ligand. Primary high-throughput screens typically look to identify low micromolar to medium nanomolar *K*_D_ value inhibitors with good assay sensitivity, the signal used to produce a clear yes/no decision through definition of an assay threshold value at three standard deviations away from the mean of negative controls. This highlights compounds for further confirmatory secondary assays and *K*_D_ value determination. Primary screens should therefore aim to use ligands which produce the largest response, through drastic reduction of a fraction ligand bound for a range of inhibitor *K*_D_ values.

For clarity, we emphasize that we are not comparing assay techniques or technologies for their establishment of traditional 3-sigma threshold values for hit calling but rather addressing a more generally applicable choice of labeled ligand affinity to maximize the assay response dynamic range, increasing the readout separation between negative and positive controls.

For the system described in Figure 1, the largest signal change for a range of inhibitor *K*_D_ values is achieved with ligand p*K*_D_ of 6.975 (105 nM *K*_D_).

To illustrate the impact of choosing a ligand with an optimal *K*_D_ value in a primary screen, we consider two situations, first using a ligand with a medium-to-high affinity of the 100 nM *K*_D_ value to the target protein and another ligand with a very high affinity of the 1 nM *K*_D_ value to the target protein. In both experiments, we hold the concentration of the labeled ligand at 10 nM and use the standard library compound screening concentration of 10 μM. The added inhibitor which we attempt to detect and infer binding through the assay readout has a target protein *K*_D_ affinity value of 1 μM. The experiments produce the following results: (i) Using a labeled ligand with a *K*_D_ affinity value of 100 nM for a target protein requires 240 nM protein to achieve a 0.7 fraction ligand in complex in the absence of an inhibitor. The addition of an inhibitor results in 0.181 fraction ligand in complex, with a readout reduction of 74.1%. (ii) Using a labeled ligand with a *K*_D_ affinity value of 1 nM for a target protein requires 9.3 nM protein to achieve a 0.7 fraction ligand in complex in the absence of an inhibitor. The addition of an inhibitor results in a 0.348 labeled ligand bound, with a readout reduction of 50.3%. Depending on assay Z-prime–and the associated assay threshold value, the hit may be missed in the assay utilizing the high affinity 1 nM ligand. Translation of the assay to use highly sensitive techniques with a reduced background, such as confocal fluorescence fluctuation analysis at single molecule resolution or time-resolved fluorescence energy transfer,^23^ may enable assays which can operate with a reduced target fraction ligand bound in the absence of an inhibitor. With a lower target fraction ligand bound, the difference in signal reduction caused by ligands of different target affinities widens. To demonstrate this, we consider two more experiments, identical to those described above, but using a target fraction ligand bound in the absence of an inhibitor of 0.1 instead of 0.7. The medium-to-high affinity 100 nM *K*_D_ value ligand and the high affinity 1 nM *K*_D_ value ligand would produce responses of 0.011 and 0.051 fraction ligands bound, respectively, in the presence of an inhibitor. This is equal to 89.2% and 48.5% of the readout’s dynamic range for each assay, further increasing the likelihood of misassigning the inhibitor as nonbinding when incorrectly applying Huang’s advice to use the highest possible affinity labeled ligand in competition-based primary screening. See Supporting Information Figure S2 for visualization of the impact that changing the target fraction ligand bound has on the detection of inhibitors. Supporting Information Figures S3, S4, and S5 illustrate the effects of changing the total concentrations of ligand, inhibitors, and finally, a matrix of plots changing the fraction ligand bound and ligand concentration. Supporting Information Figures S6 and S7 show 3D surface and contour plots, respectively, illustrating the effect of changing interactor affinities on the fraction ligand bound, while Figure S8 illustrates the behavior of a low fraction ligand bound system taken from the literature.

The maximally sensitive competition-based primary screening assay would utilize the low target fraction ligand bound, high inhibitor concentration, and a low ligand concentration, with ideal ligand affinities around the 100 nM *K*_D_ value for commonly used assay parameters.

**Figure 2:**
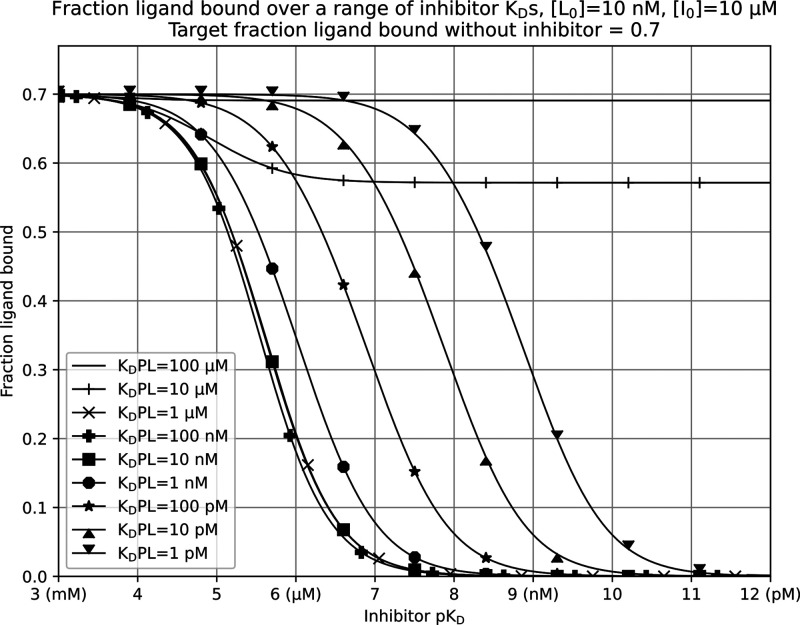
An alternative visualization of competition experiments, showing an inhibitor p*K*_D_ vs a fraction ligand bound over a range of ligand *K*_D_ values. Protein concentration appropriate to obtain a 0.7 fraction ligand bound in the absence of an inhibitor. *K*_D_PL is protein–ligand complex affinity, [*L*_0_] is the total ligand concentration, and [*I*_0_] is the total inhibitor concentration.

Figure 2 is an alternative representation of the data in Figure 1, swapping ligand p*K*_D_ on the *x*-axis for inhibitor p*K*_D_. This allows at-a-glance visualization of signal reduction for a range of inhibitor affinity values and is arguably more intuitive for practical assay readout visualization and for understanding expected responses. Within Figure 2, we can see that with a weakly binding 100 μM *K*_D_ value ligand (top solid line, without markers), a small signal reduction hardly disrupting a 0.7 fraction ligand bound is present over all inhibitor *K*_D_ values. Clearly, this is of no use in a competition assay using standard concentrations. We start to see an increased response when using a ligand with a *K*_D_ value of 10 μM to the target (line marked with +). We detect a significant signal reduction over a range of inhibitor *K*_D_ values from 1 μM (a p*K*_D_ of 6) and lower. From values of 100 nM *K*_D_ (a p*K*_D_ of 7), increased ligand affinity only serves to shift the response curve to the right, reducing the readout change for low affinity inhibitors. This 100 nM ligand *K*_D_ value limit represents the characteristic rise encountered at the right-hand side of the response valley in Figure 1.

Using simulation, we have demonstrated the impact that suboptimal ligand affinities can have in competition-based primary screens, leading to reduced assay sensitivity and fewer hits. For secondary assays, such as those used to determine exact inhibitor *K*_D_s and where Huang’s advice is intended to be applied, a strong, clear, binary yes/no response is the opposite of what is required. Here, maximal separation between inhibitors of different affinities is required. The almost binary response shown in Figure 1 using a 100 nM *K*_D_ value ligand (p*K*_D_ of 7) would make it difficult to distinguish among 1, 10, and 100 nM inhibitor affinities as the fraction ligand bound is reduced to almost zero in all cases. Huang notes that using a 100 nM *K*_D_ value ligand “will limit the high end of resolvable inhibitor potency to be roughly 100 nM”^10^ in competition experiments aiming to determine inhibitor affinities. Additionally, Figure 1 may be used to intuitively understand Huang’s advice in the context of secondary assays for *K*_D_ determination. Poor separation between inhibitor *K*_D_s is evident for low affinity ligands. The higher the ligand affinity, the more differentiable inhibitors of varying *K*_D_s are, with a large separation present between inhibitors on the right-hand upwardly sloping side of the plot denoting smaller changes in the fraction ligand bound upon introduction of inhibitors.

## Conclusion

High attrition rates in drug discovery have refocused efforts away from traditionally successful and easily “druggable” target classes. There is now a desire to “drug the undruggable” and tackle challenging targets like protein–protein interactions characterized by very high affinities, increasing performance and sensitivity requirements for primary screening assays. Application of simulation techniques outlined in this application note can contribute to the creation of more performant primary screens and help with detection of low affinity inhibitors of “undruggable” targets. Any measures taken to increase sensitivity contribute to expanding the chemical diversity of hits which may be further translated into novel leads.

We believe that application of our CLAffinity tool presented in this application note will prevent the inappropriate application of Huang’s rule which was intended for secondary confirmatory assays, not primary screening assays. These confirmatory or follow-on assays are in stark contrast to primary screens, where a clear binary yes/no may be desired indicating the detection of inhibitors over a wide range of affinities. We have demonstrated that choosing high affinity ligands can lead to the rejection of compounds with signals under unnecessarily high detection limits. This thorough understanding of competition systems allows tuning or detuning of assays toward the primary screening phase for high sensitivity or secondary screening phases involving SAR studies with detailed affinity determinations. We suggest experimental setup and planning is always based on simulations including known ligand *K*_D_ values along with detection limitations imposed by the screening technologies. We stay mindful of other assay pitfalls such as solubility and readout issues like inner filter effects which are not addressed by our simulation of perfect assay system behavior and readouts. The open source Python code used to produce all simulations and plots in this manuscript, the CLAffinity tool, and its supporting information is freely available along with tools to simulate competition experiments and aid in experimental planning at https://github.com/stevenshave/competition-label-affinity. Additionally, CLAffinity has been submitted to the Python Package Index and is therefore installable using the pip Python package manager. The Supporting Information gives a more in-depth view on the effects of changing assay system parameters. Supporting Information Video V1 provides a narrated and animated walk-through of key observations around the label affinity choice. A simple lookup table is also supplied as Supporting Information Table T1, allowing quick estimation of optimum ligand affinity for competition-based primary screens over a range of conditions.

## Data and Software Availability

Full open source Python code listings used to produce all simulations and plots in this manuscript, its supporting information, accompanying video, and the CLAffinity tool itself are freely available along with tools to simulate competition experiments and aid in experimental planning at https://github.com/stevenshave/competition-label-affinity.

## Supporting Information Available

Equation for calculation of protein amount required to reach given fraction ligand bound at given ligand concentration and affinity and plots of competition system behavior as function of changing assay parameters (PDF)
Video guide summarizing main points of interest identified in this study (MP4)
Lookup table allowing estimation of optimum ligand affinity for given range of competition assay conditions (XLSX)
