# Supplementary material for: CLAffinity: A Software Tool for Identification of Optimum Ligand Affinity for Competition-Based Primary Screens
Source: J Chem Inf Model. 2022 Apr 20;62(10):2264–8. doi: 10.1021/acs.jcim.2c00285 (PMC9131445; doi:10.1021/acs.jcim.2c00285)
Supplement: Supplementary file 1 — ci2c00285_si_001.pdf [file ci2c00285_si_001.pdf]

Supporting Information

CLAffinity; a tool for identification of optimum ligand affinity for competition-based primary screens

Steven Shave[1*], Nhan T. Pham[1], and Manfred Auer[1*]

[1] School of Biological Sciences, University of Edinburgh, The King's Buildings, Edinburgh, EH9 3BF, Scotland, UK

*corresponding authors: s.shave@ed.ac.uk, manfred.auer@ed.ac.uk

Supporting Software

Python code to perform the simulations and create plots present in the main comment text and this supporting information is available at:

https://github.com/stevenshave/competition-label-affinity

Equations to determine fraction ligand bound in competition experiments are too long to include here but can be located in the supporting software as Python functions.

Supporting Equation S1

$$P_0 = \frac{K_D . F + [L_0] . F - [L_0] . F^2}{1 - F}$$

Equation to calculate protein concentration $[P_0]$ required to achieve a target fraction ligand bound F at a given concentration of total ligand $[L_0]$ and known affinity K_D.

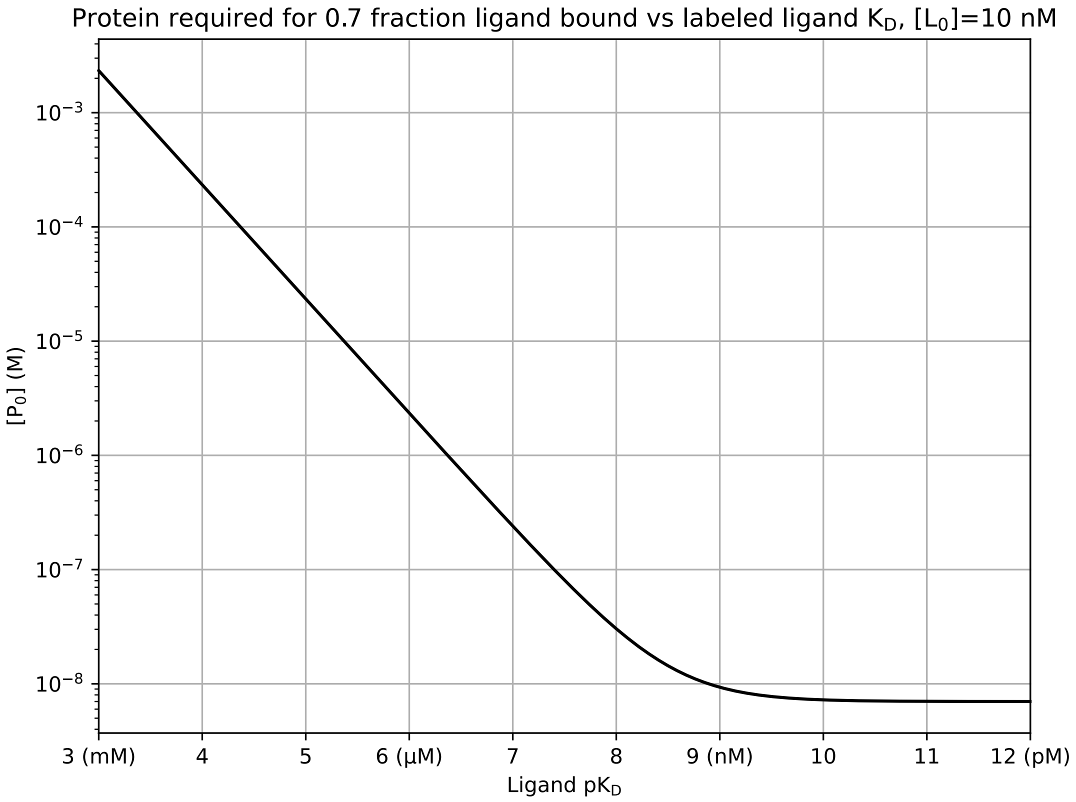

Figure S1 – Amount of protein required (P_0), to reach 0.7 fraction ligand bound for 10 nM of a given ligand with affinity expressed as a pK_D ($-\log_{10}(K_D)$).

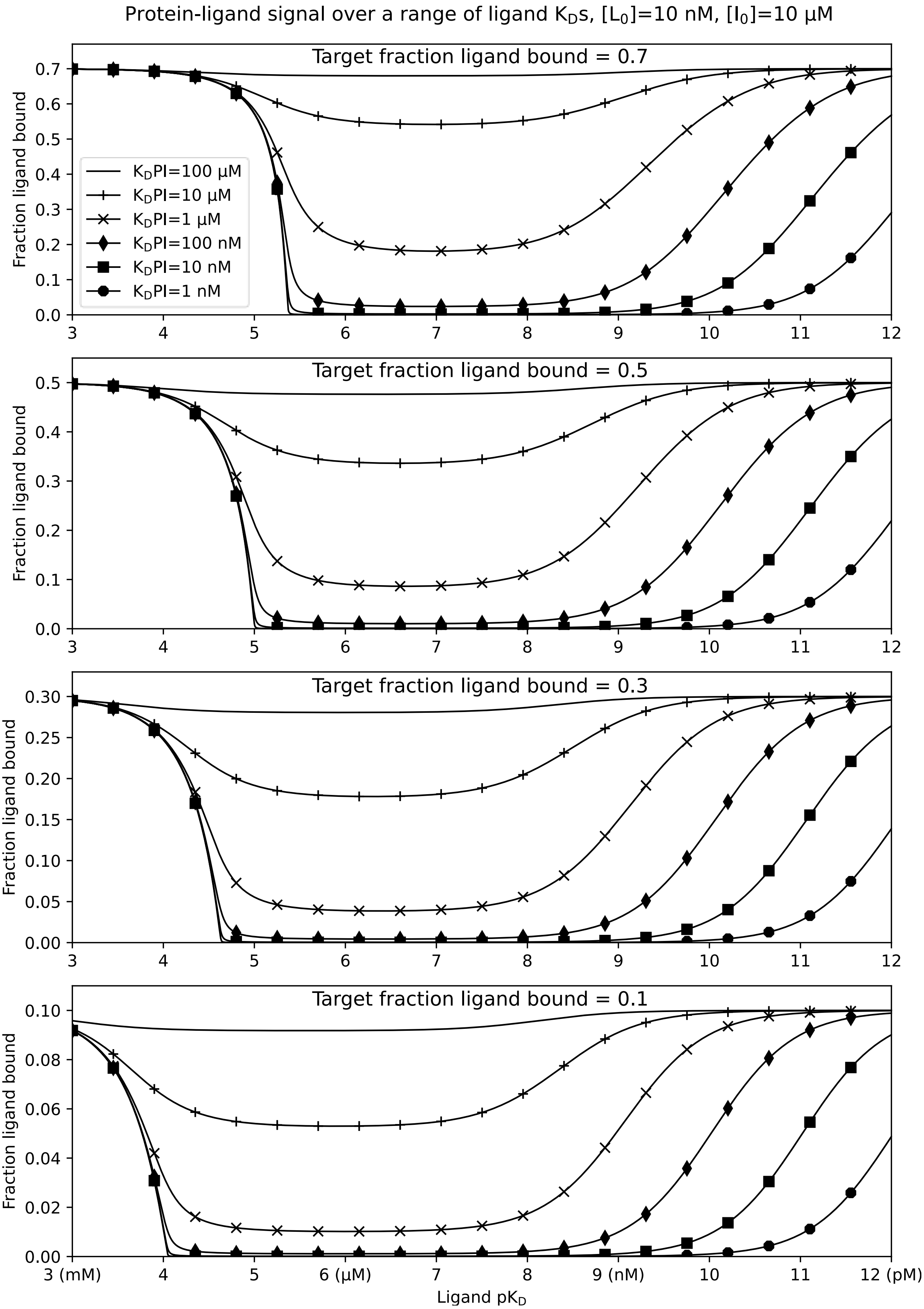

Figure S2 – The top plot is a reproduction of Figure 1 within the manuscript, demonstrating the effect of varying target fraction ligand bound (in absence of inhibitor) and how ligand affinity in competition experiments affects the detection of inhibitors over a range of K_Ds. The valley shape

characterises in the first downwards phase, the increased sensitivity which comes with higher affinity ligands, allowing the use of less protein to reach the required fraction ligand bound. Subsequent plots reduce the target fraction ligand bound, allowing the use of even less protein, and further increasing assay sensitivity to low affinity inhibitors. K_DPI is protein-inhibitor complex affinity, $[P_0]$ is total protein concentration, $[L_0]$ is total ligand concentration and $[I_0]$ is total inhibitor concentration.

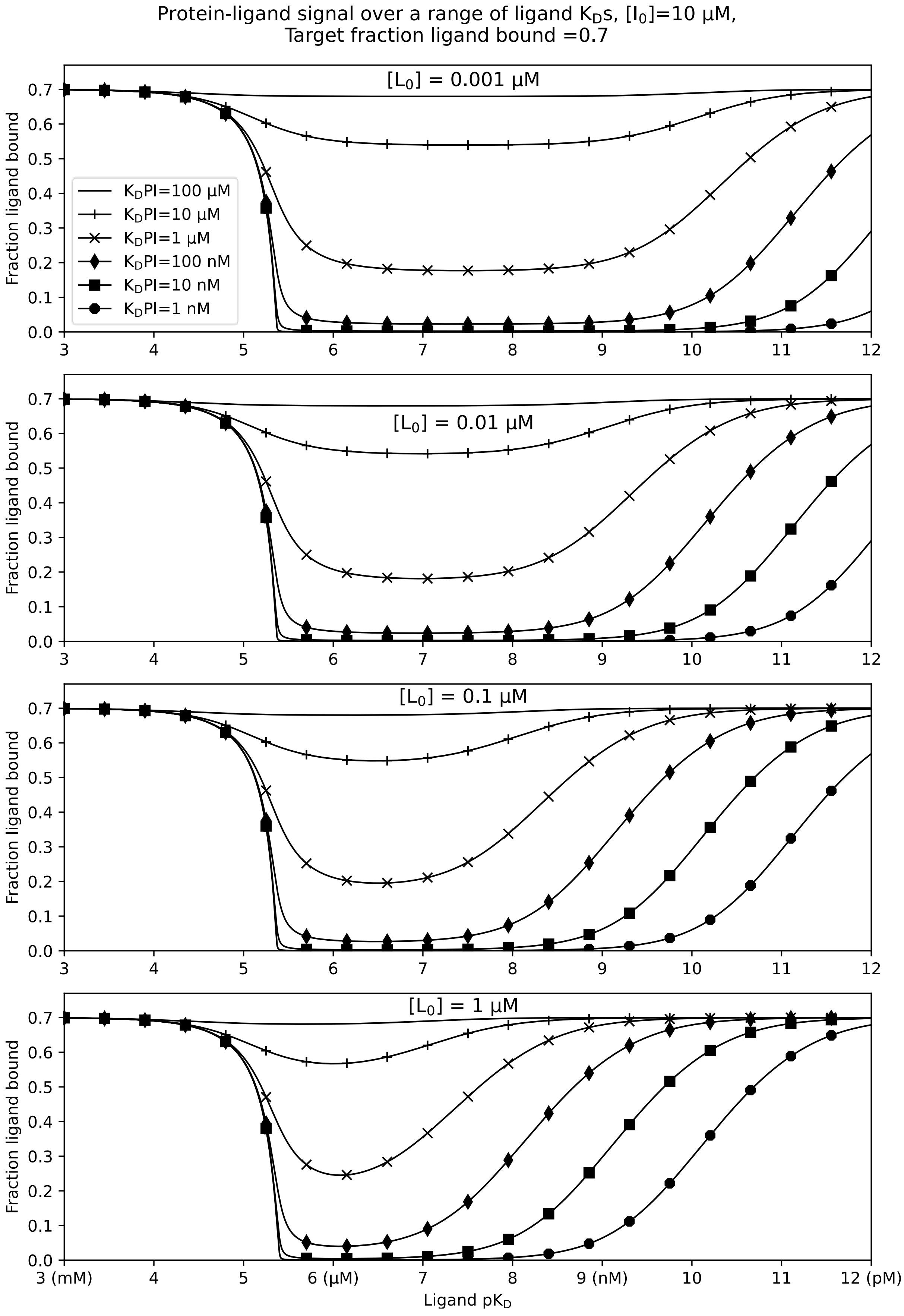

Figure S3 – The plot second from the top with a ligand concentration of 10 nM (0.01 μM) is a reproduction of figure 1 within the manuscript, subsequent plots demonstrating the effect of varying

total ligand concentration and how ligand affinity in competition experiments affects the detection of inhibitors over a range of K_Ds. Again, the valley shape characterises in the first downwards phase, the increased sensitivity which comes with higher affinity ligands, allowing the use of less protein to reach the required fraction ligand bound. K_DPI is protein-inhibitor complex affinity, $[P_0]$ is protein concentration, $[L_0]$ is ligand concentration and $[I_0]$ is inhibitor concentration. As is expected, as the amount of inhibitor present in the system increases, the assay response to all inhibitor K_Ds is reduced. Each increase in $[L_0]$ requires the use of more protein to reach the target fraction ligand bound, causing the characteristic valley of assay response to narrow, and pronouncing the impact of tight binding ligands at higher affinities. The opposite effect is seen in the top plot, where the amount of labelled ligand is reduced to 1 nM, producing a more sensitive assay, yet requiring even more precise instrumentation to read such a reduced signal.

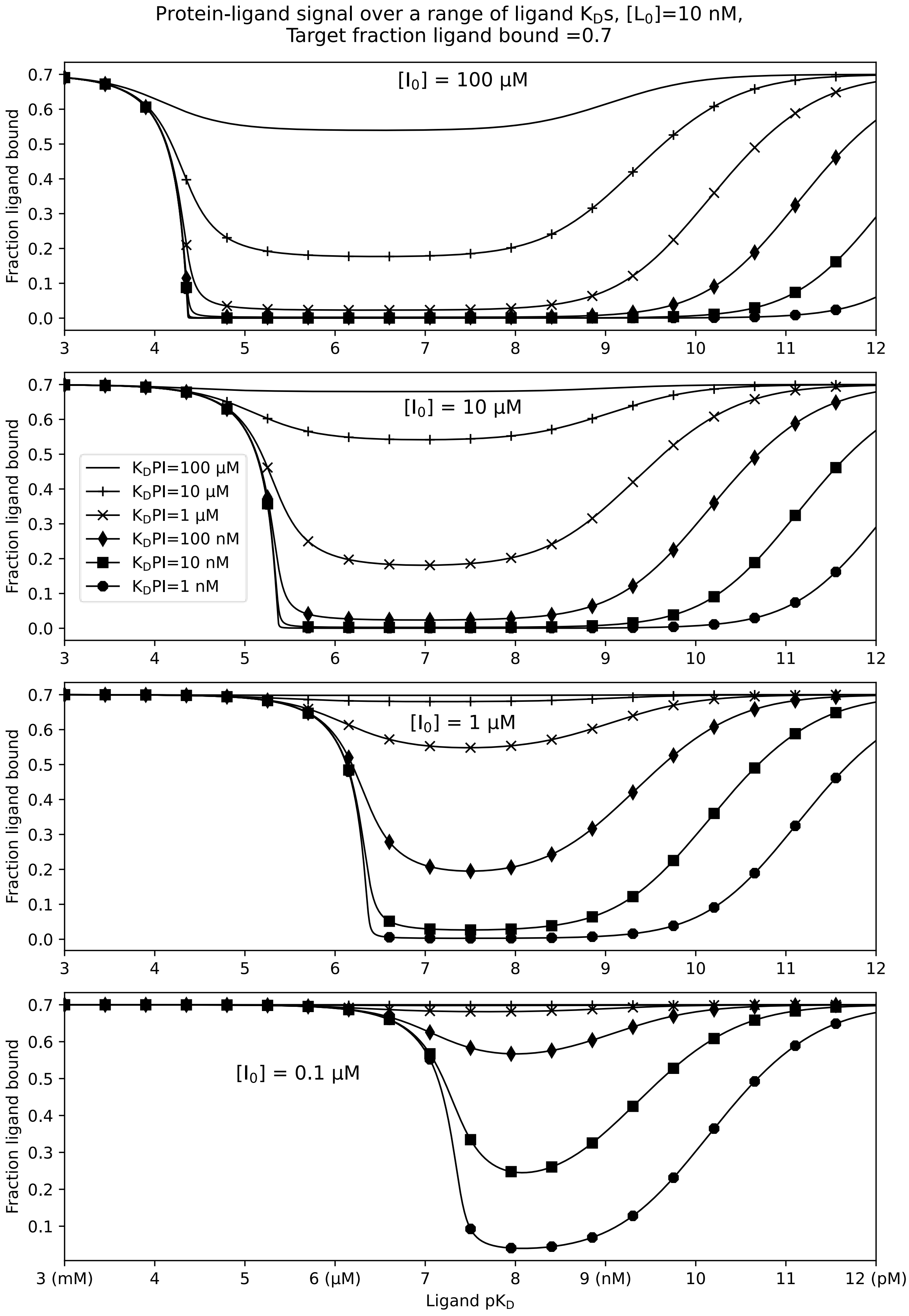

Figure S4 – The plot second from the top with an inhibitor concentration of 10 µM is a reproduction of figure 1 within the manuscript, subsequent plots demonstrate the effect of varying the total

inhibitor concentration and changing this value from the industry standard screening concentration of 10 µM. K_DPI is protein-inhibitor complex affinity, [P_0] is protein concentration, [L_0] is ligand concentration and [I_0] is inhibitor concentration. As the amount of inhibitor present is reduced, the detection range of even high affinity inhibitors is reduced, and only significantly detectable over a short range of ligand KDs around the nM range. Naturally, the top plot containing 10-times the standard screening concentration of inhibitor produces a more sensitive assay, detecting lower affinity inhibitors, producing even a small signal for inhibitors with K_Ds as high as 100 µM.

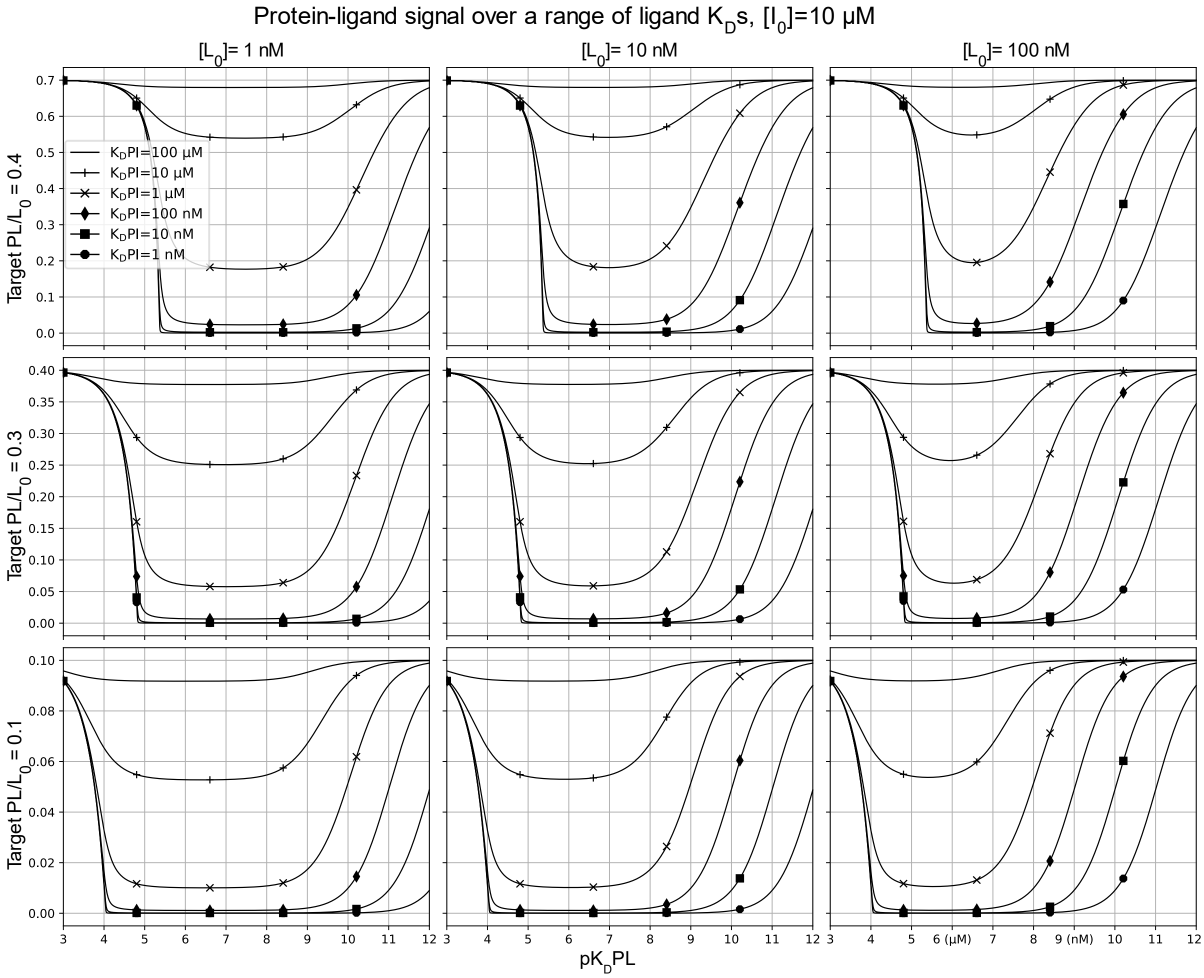

Figure S5 – Matrix of varying Target fraction ligand bound and K_DPL for individual plots profiling the effect of varying inhibitor concentration and how ligand affinity in competition experiments affects the detection of inhibitors over a range of K_Ds. K_DPI is protein-inhibitor complex affinity, $[P_0]$ is protein concentration, $[L_0]$ is ligand concentration and $[I_0]$ is inhibitor concentration. The first, second and third rows represent a target fraction ligand bound values of 0.4, 0.3 and 0.1 respectively. The first, second and third columns represent using total inhibitor concentration values of 1, 10 and 100 nM. The most sensitive assay as represented by the broadest signal valley is represented by the plot in the lower left corner, with a target fraction ligand bound of 0.1, utilising 1 nM of labelled ligand. This would represent a labelled complex concentration of 100 pM, and is impractical, yet serves to illustrate the behaviour of these competition systems and the trade-offs made between sensitivity and instrumentation.

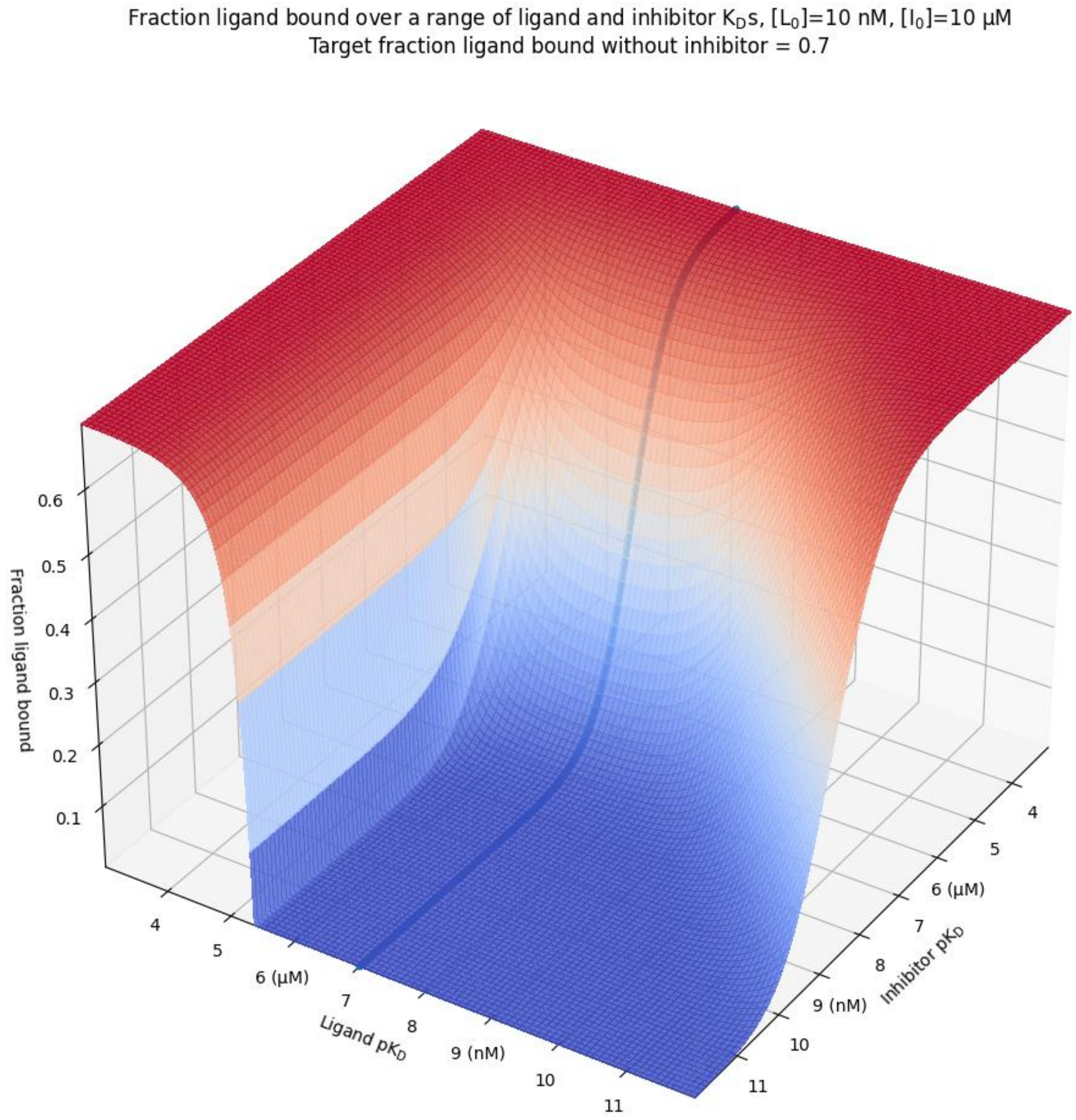

Figure S6 – 3D surface plot of fraction ligand bound over varying ligand and inhibitor affinities using a 0.7 target fraction ligand bound in the absence of inhibitor and standard ligand (L_0) and inhibitor (I_0) concentrations of 10 nM and 10 µM respectively. As can be seen from the plot, the largest change (reduction) of fraction ligand bound across all inhibitor affinities is achieved using a ligand pK_D of 6.975 (105 nM), as this value lies in the deepest part of the valley generated by the binding system (indicated by the dark blue line). Interactive exploration of the above surface plot is achievable using the Python source file “10_3D_KDPL_vs_KDPI_vs_FLB.py” in the GitHub repository accompanying this manuscript at: https://github.com/stevenshave/competition-label-affinity (visited 20/11/2021).

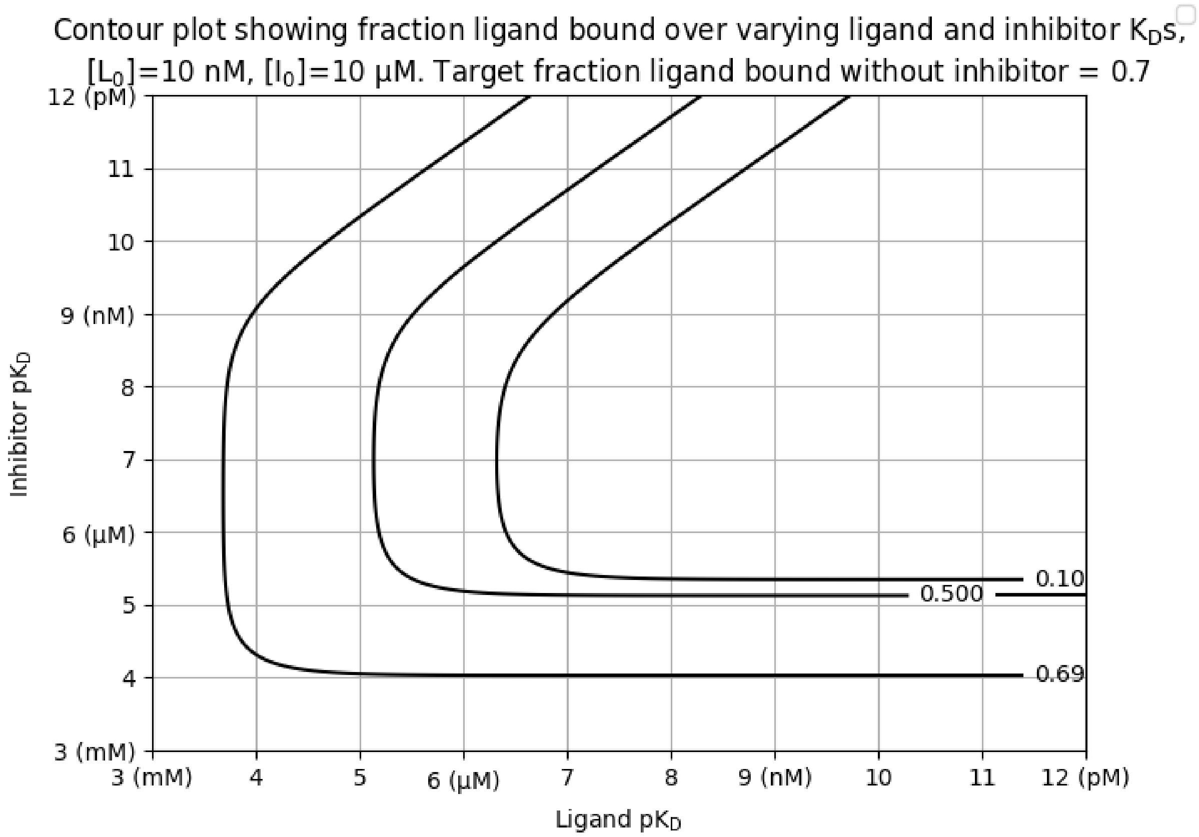

Figure S7 – Contour plot at 0.1, 0.5 and 0.69 fraction ligand bound over a range of protein and ligand affinities. $[L_0]$ = 10 nM, $[I_0]$ = 10 µM, and target fraction ligand bound in the absence of inhibitor = 0.7.

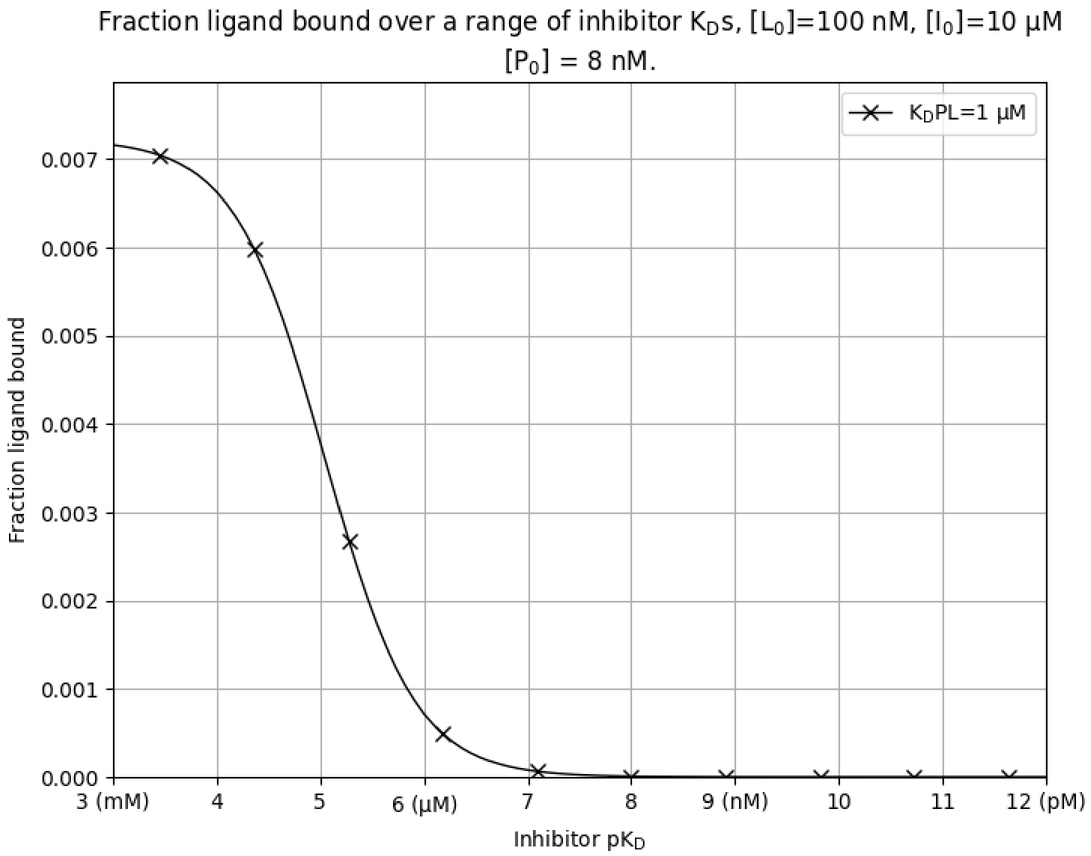

Figure S8 – Fraction ligand bound over a range of inhibitor K_D values for a highly sensitive theoretical TR-FRET experiment using 8 nM protein (P_0), 10 µM inhibitor (I_0), 100 nM ligand (L_0) with a 1 µM K_D affinity to the target protein. This system exemplifies application of the techniques outlined in the manuscript to systems operating with very small fraction ligand bound values. The system exhibits around a 50% change in fraction ligand bound with 100 µM K_D value inhibitors, resulting in a low assay threshold indicative of a highly sensitive assay able to identify low affinity inhibitors.

Supporting Video M1 – Video including an introduction to the concepts outlined in this manuscript and animated versions of Figures.

Supporting Video M1 provides a narrated and animated walkthrough of the main points of interest identified in this exploration of labelled ligand affinity choice in competition experiments.

Supporting Table T1 – Spreadsheet containing a lookup table for quick estimation of optimum ligand affinity choice for a given system

The lookup table in the accompanying spreadsheet file may be used to estimate the optimum choice of ligand for a given system without the need to perform simulation. Optimum ligand K_D translates to the largest signal across all inhibitor K_Ds.

Using the rule that the amount of protein used is calculated depending on the required fraction ligand bound, and is a function of ligand K_D and amount, the other parameters are as follows: on the data sheet, p[L0] is the negative of the log_{10}(10) of the total ligand amount, and p[I0] is the negative of the log(10) of the total inhibitor amount.

If exact values for the experimental system are not present in the table, then the closest suitable values may be chosen, or interpolation used.

Example 1
In a competition experiment targeting a 0.7 fraction ligand bound with 10 nM label and 10 µM inhibitor screening concentration, the value is read off from a TFLB (Target Fraction Ligand Bound) value of 0.7, $p[L_0]$ of 8, and a $p[I_0]$ of 5, translating to cell R40 on the data sheet, giving an ideal pK_DPI giving the maximum response of pK 6.975 = 10^-6.975 = 105.925 nM.

Example 2
In a competition experiment targeting a 0.5 fraction ligand bound with 2 nM label and 1 µM inhibitor screening concentration, the value is read off from a TFLB (Target Fraction Ligand Bound) value of 0.5, the closest (1 nM) present value for $p[L_0]$ (9), and a $p[I_0]$ of 6, translating to cell T72 on the data sheet, giving an approximate ideal pK_DPI giving the maximum response of pK_DPL 7.592 = 10^-7.592 = 25.5 nM.

Example 3
In a competition experiment targeting a 0.2 fraction ligand bound with 100 nM label and 100 µM inhibitor screening concentration, the value is read off from a TFLB (Target Fraction Ligand Bound) value of 0.2, $p[L_0]$ of 7, and a $p[I_0]$ of 4, translating to cell P113 on the data sheet, giving an ideal pK_DPI giving the maximum response of pK 5.251 = 10^-5.251 = 5.610 µM.
